# Unsupervised *Hebbian* learning experimentally realized with analogue memristive crossbar arrays

**DOI:** 10.1038/s41598-018-27033-9

**Published:** 2018-06-11

**Authors:** Mirko Hansen, Finn Zahari, Hermann Kohlstedt, Martin Ziegler

**Affiliations:** Nanoelektronik, Technische Fakultät Kiel, Christian-Albrechts-Universität Kiel, Kiel, 24143 Germany

## Abstract

Conventional transistor electronics are reaching their limits in terms of scalability, power dissipation, and the underlying Boolean system architecture. To overcome this obstacle neuromorphic analogue systems are recently highly investigated. Particularly, the use of memristive devices in VLSI analogue concepts provides a promising pathway to realize novel bio-inspired computing architectures, which are able to unravel the foreseen difficulties of traditional electronics. Currently, a variety of materials and device structures are being studied along with novel computing schemes to make use of the attractive features of memristive devices for neuromorphic computing. However, a number of obstacles still have to be overcome to cast memristive devices into hardware systems. Most important is a physical implementation of memristive devices, which can cope with the high complexity of neural networks. This includes the integration of analogue and electroforming-free memristive devices into crossbar structures with no additional electronic components, such as selector devices. Here, an unsupervised, bio-motivated Hebbian based learning platform for visual pattern recognition is presented. The heart of the system is a crossbar array (16 × 16) which consists of selector-free and forming-free (non-filamentary) memristive devices, which exhibit analogue I-V characteristics.

## Introduction

A long standing dream in machine learning is to create artificial neural networks (ANN) which match nature’s efficiency in performing cognitive tasks like pattern recognition or unsupervised learning^1,2^. Due to the impressive performance improvements of deep learning algorithms, digital computers are able to deal with complex cognitive tasks^3,4^. However, there is a drawback in terms of power dissipation and device overhead compared to the characteristic features of biological networks. The amazing performance of today’s terminal devices in cognitive tasks, such as speech recognition, is the result of skilful data sharing between the local, cost-effective device and the intelligent data processing in the “cloud”. The latter is a massive power consuming computer (server) often located in another part of the world. Although this strategy seems to be genius at first glance, severe questions appear if a local data processing (at the particular place of action) is desired, where energy supply and space is typically very limited. For example, in the future local systems of autonomously driven vehicles might be preferable to avoid external cyber attacks on million of cars simultaneously. This data security argument also holds for biomedical applications. For respecting the patient, sensible data should not be spread out to everybody but should be handled locally and reasonably. All this motivate a paradigm shift in the research of ANN and initiated a new pathway towards analogue, in-memory computing architectures for so called, energy efficient and compact neuromorphic circuits^5,6^.

Neuromorphic engineering uses analogue VLSI (Very Large Scale Integration) based on CMOS (Complementary Metal Oxide Semiconductor) technology^5,6^. This allows the creation of real-time computation schemes in a parallel and energy-efficient architecture. These systems can be expected to handle the highly complex neural network connections better than serial computational schemes^6^. However, the energy efficiency of neuromorphic systems is decidedly determined by realization of the interconnections between artificial neurons (synapses). The implementation of synaptic functionalities needs non-volatile devices, which emulate the analogue and plastic learning behaviour of synapses in an integrated circuit. In this context, memristive devices offer unique perspectives for neuromorphic circuits due to their low power consumption and the high integration density of the devices on a chip^7–9^. Moreover, memristive devices enable the emulation of synaptic functionality in a detailed and efficient manner^8–15^. So far, important local biological synaptic mechanisms have been realized, such as Hebb´s learning rules^10^ including spike-timing dependent plasticity (STDP)^8,16^, long-term potentiation (LTP) and its counterpart long-term depression (LTD)^17^. Due to this unique behaviour of memristive devices, promising computing schemes, including implicit learning schemes^18,19^, auto-associative networks^20,21^, and perceptron networks for pattern recognition^22,23^, classification^24–27^, and unsupervised learning^28–30^, have been already presented.

However, a gap remains between these promising computing schemes and their hardware realization. In particular, the integration of the memristive devices with a silicon based analogous VLSI technology is a great challenge. This applies both to CMOS manufacturing processes and to the electrical signals used in the data processing schemes. Furthermore, the electrical adaptation of memristive crossbar memory arrays in conjunction with a suitable neural computing scheme have to take the inherent limitations of the memristive devices (variability, reliability, and dynamic range) into account ^31,32^. Although considerable progress has been made in the last few years in this field, a variety of challenges remains. This includes the need of memristive devices with a high I-V non-linearity and asymmetry to avoid additional selector devices for each cell of the crossbar array^31–35^. Another obstacle is the lack of electroforming-free and analogue, crossbar-integrated memristive devices to fully use the advantages of memristors over conventional CMOS technology.

In this investigation we will demonstrate memristive crossbar arrays (16 × 16) that require no selector devices and do not need initial electroforming steps. In other words, such as-fabricated crossbar arrays are “*plug and play*” units. Furthermore, these filament-free devices feature truly analogue, synaptic-like resistive switching without special writing schemes. In those devices an ultra-thin memristive layer is incorporated in between a tunnel and a Schottky barrier with the benefit that the tunnel barrier limits current through the device. The resistance switching takes place at the insulator-metal interface (Schottky contact), where the ion motion under applied electrical fields lead to a variation of the energy barrier of the Schottky contact^36–38^. These memristive devices are used as hardware synapses together with software neurons in a mixed signal circuit which allows unsupervised Hebbian learning of visual patterns.

## Results

### Crossbar integration and device characteristics

**Figure 1:**
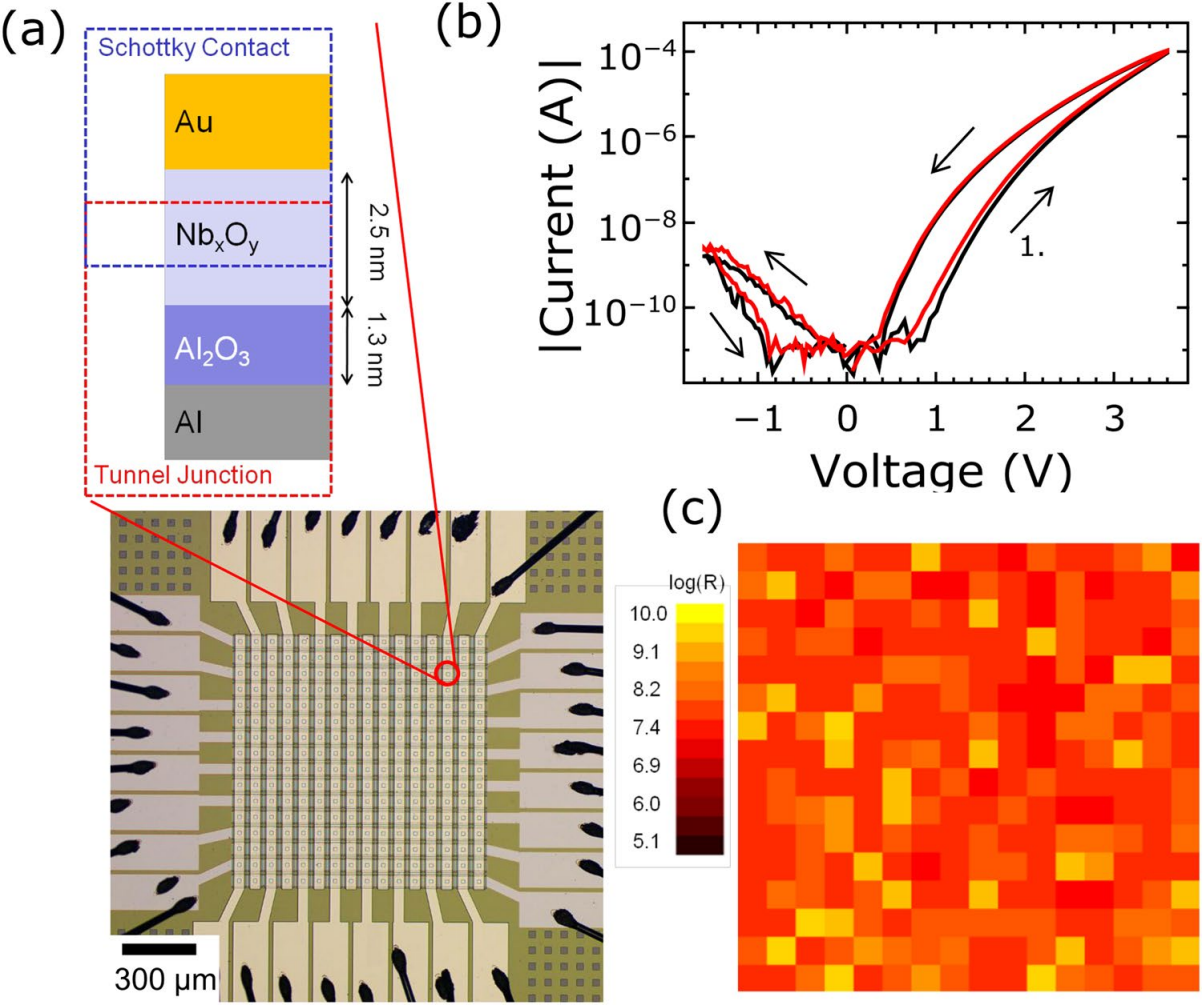
Double-barrier memristive device and crossbar integration: (**a**) Schematic cross-section of the Al/Al_2_O_3_/Nb_x_O_y_/Au double-barrier memristive device. Those devices have been implemented physically into a 16 × 16 crossbar array consisting of 256 single cells. (**b**) Comparison of I-V curves for a crossbar-device and an individual device from different wafers (for crossbar measurement, the other cells of the array were floated). To visualize the obtained change in resistance better the absolute value and a logarithmic scale was used. (**c**) Resistance map of one crossbar containing the initial resistances of 256 memristive cells measured at 0.9 V.

Figure 1(a) shows the layer sequence of the used memristive cells together with its physical implementation into a 16 × 16 crossbar array consisting of 256 single cells. In the double barrier device structure an ultra-thin memristive layer (Nb_x_O_y_) is sandwiched between an Al_2_O_3_ tunnel barrier and Au layer. The Nb_x_O_y_/Au interface can be described as a Schottky like contact^36^. Figure 1(b) shows the similarities in |I|-V curves between a crossbar device (red) and an individual cell from a different wafer. In this measurement, the voltage has been ramped from 0 to 2.8 V (cf. Fig. 1(b)) to set the device from its initial high resistant state (HRS) to a low resistance state (LRS). Afterwards, the voltage has been ramped down to −1.4 V (cf. Fig. 1(b)) and back to 0 V to reset the device. The prominent features are a distinct |I|-V non-linearity and an asymmetry between positive and negative bias, which are due to the diode characteristic of the device. The uniformity of the device resistances within the crossbar array is shown in Fig. 1(c). Here the initial resistances of the 256 junctions of the crossbar array, measured at a read voltage of 0.9 V, are shown.

The underlying physical mechanism of the device behaviour has been described in detail in^36–38^ and is based on the movement of oxygen ions within the Nb_x_O_y_ layer under the applied electric bias field. Under positive bias voltages, the electric field across the Nb_x_O_y_ layer ensures that negatively charged oxygen ions drift towards the Au interface. This results in a decrease of the Schottky barrier height as well as in an increase of electron injection through the tunneling barrier due to a decrease of the Au/Nb_x_O_y_ interfacial potential. This mechanism provides several advantages compared to single barrier devices. Chemical diffusion barriers for the ions, a defined interfacial potential, and a homogeneous resistance change rather than a binary switch observed by filamentary based devices. This decreases the device variability and improves the retention characteristics compared to single barrier devices. Furthermore, due to the Schottky-like interface, voltages below 1 V cannot affect the ions, allowing a non-destructive read-out of each cell and a selector device free addressing in the crossbar structure, as we will discuss in more detail below.

### Emulation of neural plasticity

Learning in biological networks is manifested at the cellular synaptic level, where the connectivity between neurons varies in respect to their level of activity^39,40^. This process is called synaptic plasticity and it induces a long-lasting increase or decrease of synaptic connections, so-called long term potentiation (LTP) or long-term depression (LTD)^41^. For the implementation of LTP or LTD, mainly two aspects are important: first, the level of activity of the pre- and the post-synaptic neurons and second, the relative timing of their activities, known as *spike-timing-dependent plasticity* (STDP)^16,42^. A variety of concepts have been presented in the last couple of years which use memristive devices to emulate synaptic plasticity^43^. In the following the concept applied here to emulate neural plasticity is described.

**Figure 2:**
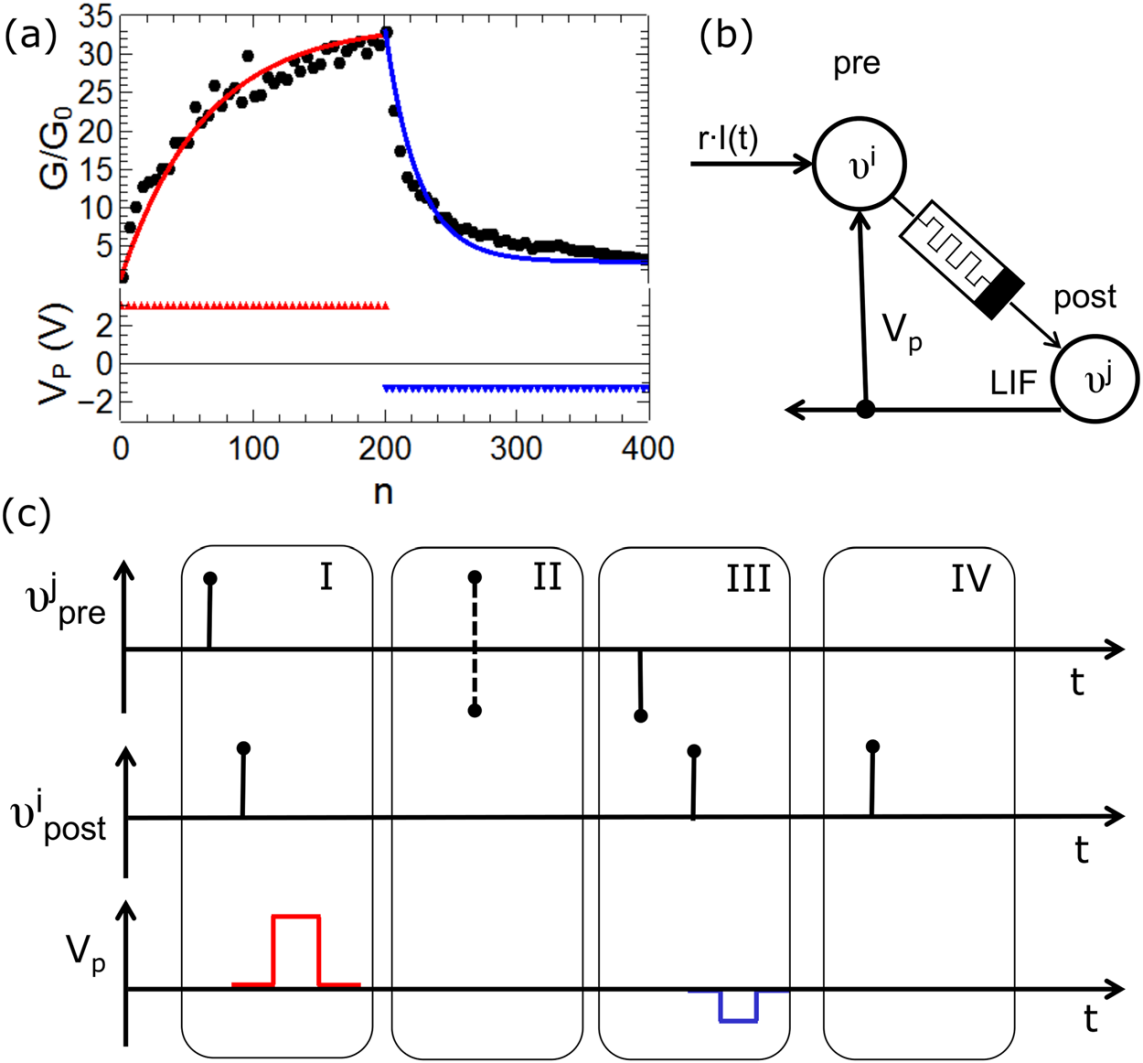
Emulation of neural plasticity. (**a**) A sequence of 200 potentiation pulses of +3.3 V with a pulse length of 100 ms and 200 depression pulses of −1.1 V with a pulse length of 300 ms (upper graph). Black dots are experimental data measured at 0.9 V, while red and blue lines correspond to the data obtained using Eq. 1. (**b**) Illustration of one memristive device connecting a pre- and a post-neuron. For the pre-neuron, a stochastic coding scheme is used which decodes the input signal intensity *S(t)* either into a positive input activity *υ*^*j*^ = +1, or a negative activity *υ*^*j*^ = −1. As post-neuron, a leaky integrate-and-fire neuron is used. (**c**) The implemented *Hebbian* learning formalism: a potentiation pulse *V*_*p*_ is applied in case (I), where the activity of the pre- and post-neuron is positive. No voltage pulse is applied to the memristive device in the cases (II) and (IV), since here is either the pre- or the post-neuron active. The dashed lines in (II) shall indicate that for the pre-neuron *υ*^*j*^ can either be +1 or −1. A depression pulse *V*_*d*_ is applied in case (III), where the pre-neuron activity is negative, while the post-neuron is active.

Figure 2(a) shows the gradual change of the device conductance of a single memristive cell under a voltage train with *n* voltage pulses *V*_*p*_. The voltage train consists of 200 equivalent positive voltage pulses (potentiation pulses of Δ*V* = 3.3 V and Δ*t* = 100 ms in red) followed by 200 equivalent negative voltage pulses (depression pulses of Δ*V* = −1.1 V and Δ*t* = 300 ms in blue). To measure the device conductance, a read voltage of 0.9 V (well below the threshold voltage of the device) was applied and the current was measured after every potentiation/depression pulse. The obtained conductance values *G*(*n*) were normalized by the average initial conductance *G*_0_ for a better illustration. We found a gradual change of the device conductance up to 3300% and a saturation of the conductance which bound *G* between *G*_0_ and a maximal conductance *G*_*max*_. To further quantify the conductance variation, the experimental data has been fitted by

1 $${G}_{n}=\{\begin{array}{cc}{G}_{n-1}\cdot (1-{{\rm{e}}}^{-{{\rm{\beta }}}_{{\rm{p}}}{\rm{\Delta }}t})+{G}_{0} & \text{for}\,\text{potentiation}\,\\ {G}_{n-1}\cdot {{\rm{e}}}^{-{{\rm{\beta }}}_{{\rm{d}}}{\rm{\Delta }}t}+{G}_{0} & \text{for}\,\text{depression}\end{array},$$

where *β*_*p*_ and *β*_*d*_ are positive constants which describe the experimental characteristic best for *β*_*p*_ = 3.4 s^−1^ and *β*_*d*_ = 0.13 s^−1^ (cf. solid lines in Fig. 2(a)) and emphasize the |I|-V non-linearity and self-saturation characteristics of the double-barrier memristive device particularly.

As a first step to integrate double barrier memristive devices into a network structure, we discuss how a single memristive device is connected to a pre- and post-neuron. This configuration is sketched in Fig. 2(b). For the pre-neuron, a stochastic coding scheme is used in which the pre-neuron decodes the probability of a spike generation depending on the input signal intensity *S(t)*^44,45^. For this purpose, the input signal is normalized to the interval [−1, 1] and in each time step a random number *r ϵ* (*0*, *1*) is generated to calculate the activity of the pre-neuron *υ*^*j*^. The pre-neuron is active for |*S(t)*| > *r(t)*. The generated pulse is positive for *S(t)* > *0* (*υ*^*j*^ = +*1*) and negative for *S(t)* < *0)* (*υ*^*j*^ = −*1*). As post-neuron, a leaky integrate-and-fire neuron is used, where a membrane capacitance *C* is connected in parallel to a conductor *g*_*L*_. They are driven by an input current *I(t)* according to

2 $$C\cdot \frac{du(t)}{dt}=-{g}_{L}\cdot u(t)+I(t)$$

Here, *u(t)* is the membrane potential. The application of an input current causes an integration of *u(t)* up to the threshold potential *θ*_*thr*_, at which the activity of the post-neuron *υ*^*i*^ is set from 0 to +1.

We used the Hebbian learning formalism to get a local learning condition which appropriately adjusts the device conductance in a network environment. Accordingly, the conductance *G* is changed whenever the pre- and the post-synaptic neurons are simultaneously active (cf. Fig. 2(b)):

3 $$\frac{dG}{dt}=\alpha \cdot {\nu }^{j}{\nu }^{j}.$$

Here, *α* denotes the local learning rate of the conductance update, which can be derived from Eq. 1 for the double barrier device by using *t* = *nΔt*. For potentiation, *α* is given by *α* = *G(t*−*1) β*_*p*_ exp*(−β*_*p*_ *Δt)*, while in the case of depression *α* reads *α* = *G(t−1) β*_*d*_ *(1−*exp*(−β*_*d*_ *Δt))*. The difference between depression and potentiation is defined by the sign of the product *υ*^*j*^*∙υ*^*i*^. Thus, in total four different cases are possible, as illustrated in Fig. 2(c). In case (I), the pre-synaptic activity is positive, while the post neuron is active as well. In this case, a potentiation pulse *V*_*p*_ is applied (cf. bottom panel in Fig. 2(c)). Case (II) corresponds to the condition where only the pre-synaptic neuron is active. In this case, no change in the device conductance is generated. Further, (III) defines the case when the pre-neuron’s activity is negative while the post-neuron is active. In this case, a depression pulse *V*_*d*_ is applied which decreases the device connectivity (cf. bottom panel in Fig. 2(c)). If the post-synaptic neuron is active without pre-neuron activity, the device conductance is not affected (case (IV)).

Equation 2 and the described learning conditions have been implemented in a combined software-hardware scheme. The activities of the neurons are calculated on a digital computer connected to a microcontroller. This addresses the single memristive cells within the crossbar array, as it will be described in the following in detail. We would like to emphasize that the digital computer is not necessary, and was only used in this proof-of-principle investigation to assess the usability of the memristive crossbar arrays.

### Network structure

**Figure 3:**
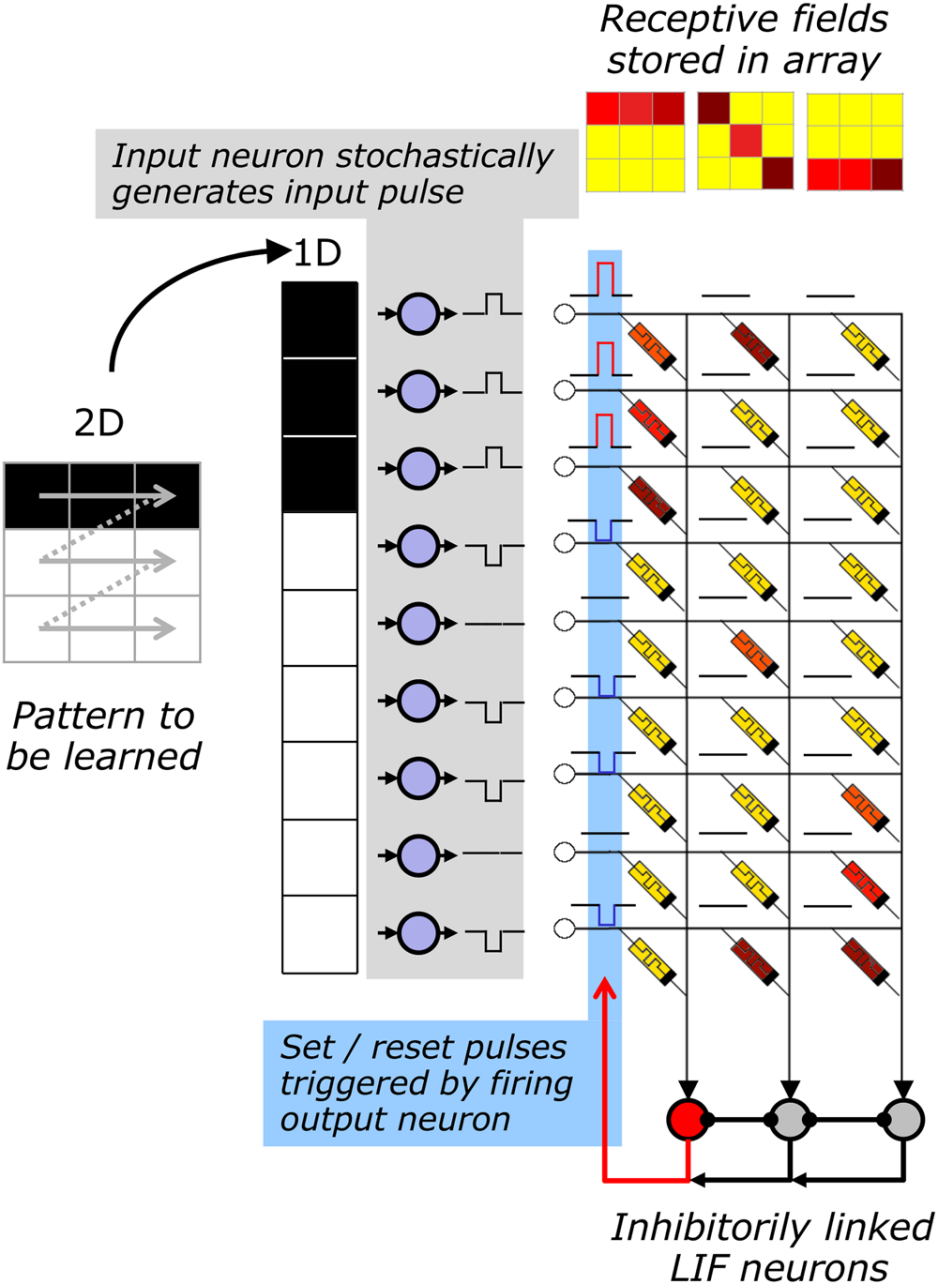
Schematics of the implemented neural network: The network consists of a stochastic coding scheme of the input data, leaky-integrate-and fire output neurons (LIF), which are laterally coupled in an inhibitory winner-take-it-all network (WTA), and memristive devices which are arranged in a crossbar structure. Receptive fields are a result of a Hebbian learning scheme.

The network structure is illustrated in Fig. 3. The network consists of two neural layers connected via an array of memristive devices into feed-forward direction. As input signal for the pre-neurons pixel values of 2-dimensional gray-scale matrices are used, which have been transformed, prior to their application, into a 1-dimensional vector. The individual pixel values define the input intensity *S*(*t*) for the pre-neurons, which stochastically generate the neural activity patterns of the input-layer according to the computation scheme explained above.

Leaky integrate-and-fire neurons are used as post-neurons in the output layer. In addition to the above described neuron model, the output neurons are laterally coupled within an inhibitory winner-takes-it-all network, including adaptive thresholds for the spiking, as proposed in^44^. The winner-takes-it-all approach is necessary to allow unsupervised learning within the network structure, in which the first spiking neuron resets the integration of all other neurons. The adjustable neuron firing threshold is crucial for unsupervised learning, because it guarantees that all output neurons participate equaly in the learning phase. This can be motivated by considering the process of homeostasis in biological systems^44–46^. Therefore, the firing threshold of a neuron is increased whenever the spike number (activity) of a neuron is above the desired activity, and vice versa. This can be achieved by using

4 $$\frac{{{\rm{dV}}}_{{\rm{th}}}}{{\rm{dt}}}={\gamma }_{{\rm{th}}}({{\rm{A}}}_{{\rm{avg}}}-{{\rm{A}}}_{{\rm{tar}}})$$

for the threshold voltage adaptation. Here, *γ*_*th*_, *A*_*avg*_, and *A*_*tar*_ are respectively the gain factor, the mean activity of an individual neuron, and the target activity *(γ*_*th*_ = 0.01 and *A*_*tar*_ = 2 during an interval of 60 images).

The Hebbian learning scheme of Eq. 3 is employed to increase or decrease the device conductance. This results in the following back-propagation procedure: if one of the post-neurons spikes, then a voltage pulse is applied to the respective memristive cells which connect the active pre- and post-neurons. In the case of *υ*^*j*^ = +*1*, a potentiation pulse is generated, while for *υ*^*j*^ = −*1*, a depression pulse is initiated (cf. the blue filed field in Fig. 3). Thus, every output neuron creates its own specific receptive field during learning (cf. sketch in Fig. 3). In the recognition phase, the neurons are able to spike in accordance with the previously learned pattern for varying input signals.

**Figure 4:**
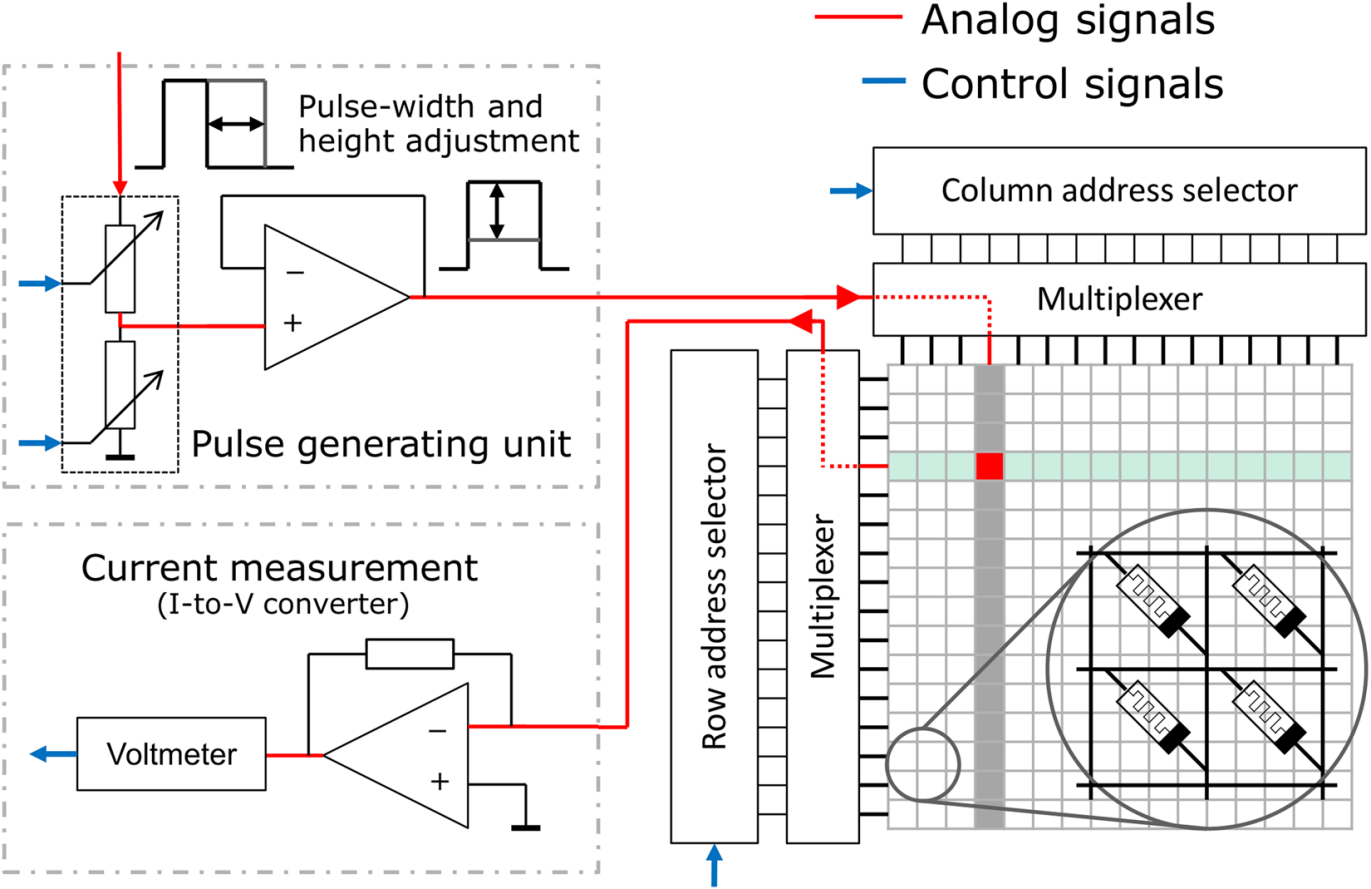
Technical realization: The crossbar array composed of memristive devices is connected through wire bonds to a custom PCB sample holder. Different sets of analogue switches allow to address a specific device. During the measurement, a single cell from the crossbar array is measured while all other cells in the crossbar array are left floating.

The neural network scheme has been implemented on a custom-made printed circuit board, as sketched in Fig. 4 and described in the method part. It consists of hardware synapses, i.e. memristive devices, and software neurons.

### Formation of receptive fields

**Figure 5:**
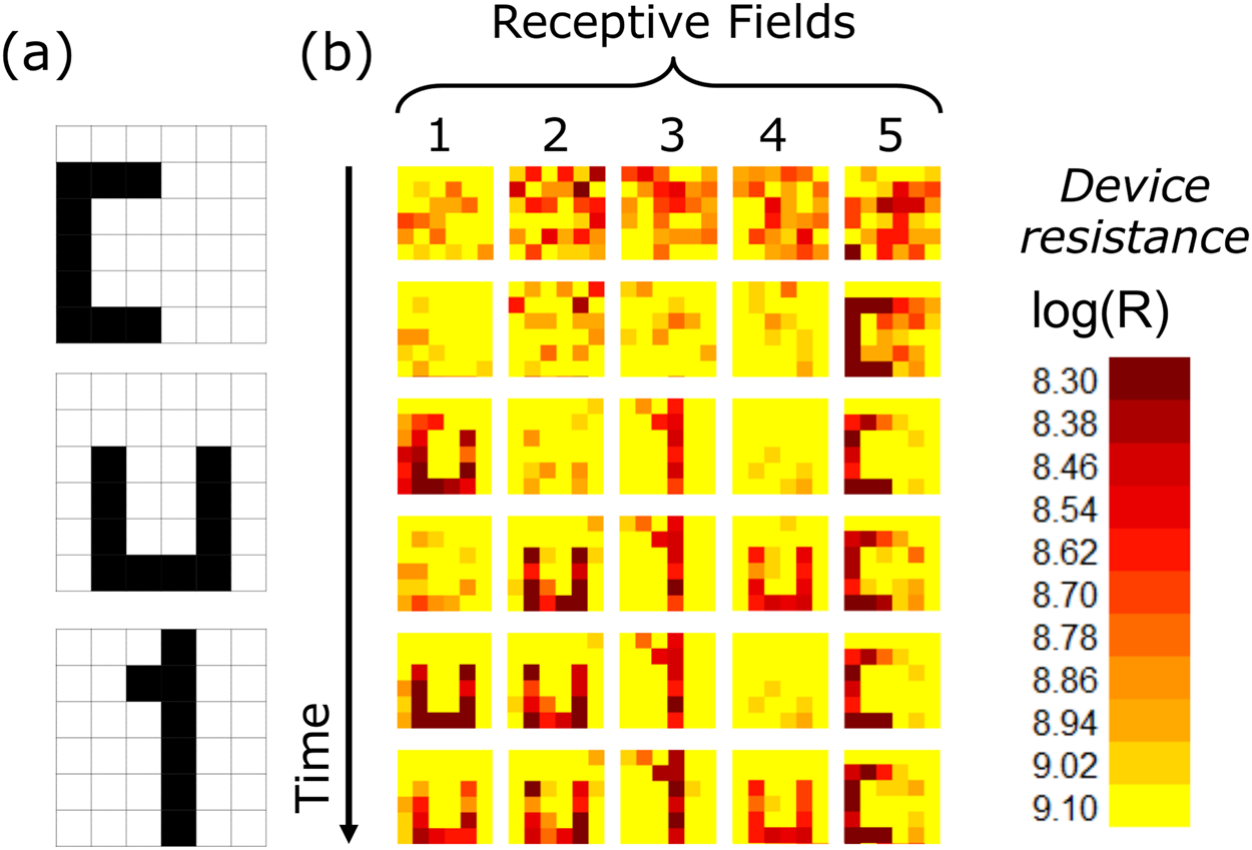
Formation of receptive fields: (**a**) Used training patterns. (**b**) Obtained receptive fields during unsupervised learning in the case of 5 output neurons. The pixels correspond to the resistance values (synaptic weights) of the memristive devices. The used parameters for the learning algorithm are listed in Table 1.

To study the formation of receptive fields, the network has been trained with three training patterns, which are shown in Fig. 5(a). The used parameters for the learning algorithm are listed in Table 1. Each of the patterns is a 6 × 6 pixel image with two colour values (black and white). Therefore, the input layer contains 36 single neurons. Five output neurons were provided, using 180 memristive devices (5 × 36) out of 256 devices in the crossbar array. To investigate the learning performance of the network, the patterns were applied in total 22,000 times to the network. Voltage pulses with different parameters were used for the potentiation (Δt = 100 ms, ΔV = 3.6 V) and depression (Δt = 300 ms, ΔV = −1.1 V). All other parameters are summarized in Table 1. We would like to emphasize, that the crossbar arrays are passive and do not require on-chip selector devices. This is possible, because the strong I-V non-linearity (cf. Fig. 1(b)) prevents interference with neighbouring devices. This can be understood regarding the I-V curve shown in Fig. 1(b): an increase in pulse height from 2 to 2.8 V leads to an increase in device conductance by a factor of 10, while below 1 V the device conductance remains unaffected. This asymmetry ensures that the electrical field for ion drift is sufficiently low in neighbouring devices during the set and reset process. Hence, the conductance of these neighbouring devices do not change. Moreover, in accordance to the non-filamentary switching mechanism, no initial forming procedure was necessary which facilitated the application of the crossbar arrays significantly.

**Table 1:** Parameters for the learning algorithm.

Parameter	Value
Neuron Model	
C	30 µF
R	400 kΩ
V_th_initial_	1 mV
t_refractory_	4 Iteration
Voltage Pulses	
V_set_	3.6 V
V_rest_	−1.1 V
dt_set_	100 ms
dt_reset_	300 ms
Current compliance	I_CC_ = 98 µA

In Fig. 5(b) the development of the receptive fields during the learning phase are shown. For this purpose, six characteristic instances in time were selected, showing the unsupervised learning mechanism. In the beginning, the conductance values of the memristive cells are randomly distributed (cf. row 1 in Fig. 5(b)). While the patterns “1” and “C” have formed quite fast in the receptive fields of the post-neurons 3 and 5, the receptive fields of the other post-neurons have been adapted by the network, so that pattern “U” was learned as well. The adaptation can be seen at the receptive field of the post-neuron 1, which learns pattern “U” (cf. first column in Fig. 5(b)). While first a mixture of pattern “C” and “U” is represented in the receptive field of this neuron, both patterns disappear and finally the pattern “U” appears in the final configuration.

After the learning phase, the network is able to distinguish the learned patterns. The back propagation is suppressed during the recognition phase, i.e. no set and reset pulses are initiated. For the case shown in Fig. 5(b) this means: post-neurons 1, 2, and 4 are active for pattern “U”, post-neuron 3 is active for pattern “1”, while neuron 5 is active whenever pattern “C” is applied as the input.

## Discussion

Crucial for machine learning schemes are their storage and discrimination capabilities, which allow them to be used in complex classification tasks. On the one hand, distinguishing similar patterns is important, since it enables an adequate response of the system in multi-disciplinary tasks. On the other hand, it is required that the system is robust against variations in input patterns, which ensures an accurate performance if the input data is noisy or incomplete. In fact, this is a rather general property of learning and the formation of memory in the brain^47^, this is why it has attracted an intense research interest in the field of neuroscience over the last decades. Two cognitive functions have been identified to be particularly important: pattern separation and pattern completion^48,49^. Pattern separation, whereby similar inputs are stored in non-overlapping and distinct representations, can be regarded as a mechanism which balances the network against pattern completion.

**Figure 6:**
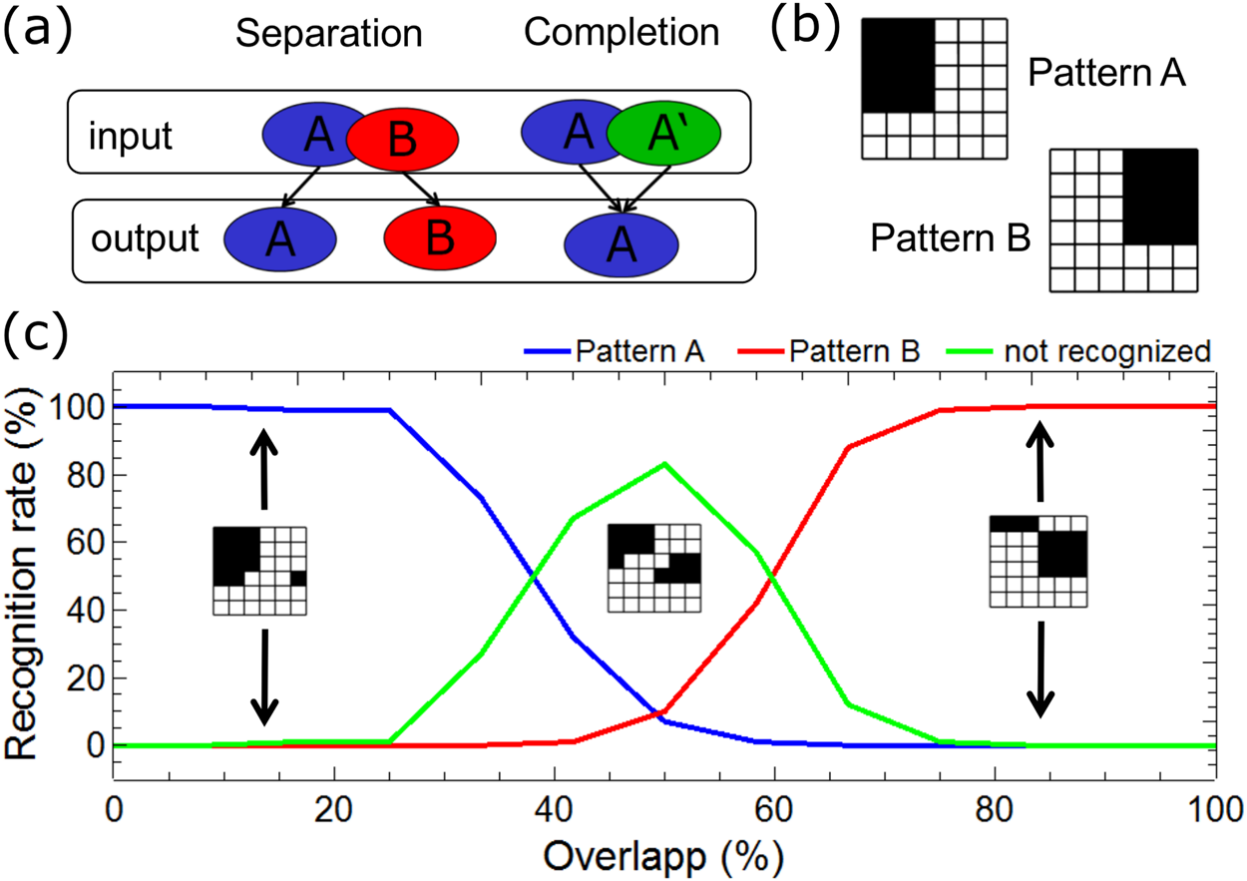
Input-output transfer characteristic of the network: (**a**) Conceptual representation of pattern separation and pattern completion as key cognitive functionalities which underline the process of pattern recognition. (**b**) Patterns used to train the network. For these two non-overlapping patterns, a network with 36 input neurons and 2 output neurons is used. (**c**) Recognition rates of the network depending on the strength of the overlap of patterns A and B.

The function of pattern separation and pattern completion within the here presented network has been investigated and the obtained results are shown in Fig. 6. In Fig. 6(a), a conceptual illustration of pattern separation and pattern completion is given, which describes the transfer behavior of information through the network: pattern completion increases the overlap of the representation of two patterns (labeled as A and B in Fig. 6(a)), while the process of pattern separation reduces the overlap. The obtained transfer characteristic of the here implemented network has been evaluated by using the two non-overlapping patterns shown in Fig. 6(b). The transfer characteristic which is obtained therewith is shown in Fig. 6(c). Here, the recognition rates as functions of the overlap between the two input patterns (A and B) are plotted. The recognition rates are defined as the spike intensities of the post-neurons during the recognition phase. For this purpose, only two neurons are used and the spiking dynamics under a varying input of those two output neurons are investigated. Furthermore, the response of the two outputs is digitalized, so that either pattern A (blue curve in Fig. 6(c)) or pattern B (red curve in Fig. 6(c)) has been recognized if neuron 1 or neuron 2 is active, respectively. The input has not been recognized (green curve in Fig. 6(c)) if neither neuron 1 nor neuron 2 is active or they are both active together. In total, the input patterns are applied 14,000 times. We found a recognition rate of 100% if the number of replaced pixel is below 25%. However, when half of the patterns A and B are applied at the same time, a marked increase in the non-recognition rate is observed. This case might be interpreted that neither pattern A nor pattern B has been recognized. In this case the system might regard the input as new, i.e. as a pattern which has not been learned during the training phase.

In order to account best for a possible variability within the input patterns for this computing scheme the number of output neurons can be increased. This point has been addressed in a couple of theoretical investigations^44,46,50^. It has been found that recognition rates up to 93.5% are possible with such a simple two-layered network. However, those networks need ≈235,000 memristive cells, which poses strong requirements on the device variability^44^. In this respect, our double-barrier memristive device has a suitable variability as we were able to show recently^51^.

In conclusion, an unsupervised learning scheme for pattern recognition has been implemented in a mixed signal circuit consisting of analogue hardware synapses and digital software neurons. For this purpose, memristive double barrier devices are integrated into a crossbar array structure, which contains 256 single memristive cells. The strong I-V non-linearity and asymmetry of the individual cells has been used to implement associative Hebbian learning in a selector device free crossbar configuration. Particularly, this enables the realization of a local learning scheme for the formation of receptive fields. The transfer characteristics of the implemented circuit have been used to analyze their capability for pattern separation and pattern completion. At this respect, the crucial factors are the storage and discrimination capabilities of the network, which are restricted to the number of memristive cells in the crossbar array. The presented system is in principle able to cope with more complex tasks, with the drawback of a larger demand of memristive cells. Due to the large HRS and LRS in comparison to the individual wiring resistance (≈100 *Ω* for an individual wire with the size 1,100 × 40 × 0.5 *µm*^3^) in the present crossbar we expect that larger (m × n) arrays will function as well.

## Methods

### Sample preparation

Memristive tunneling junctions were fabricated on 4-inch Si wafers with 400 nm of SiO_2_ (thermally oxidized) using a standard optical lithography process. The devices were fabricated using the following procedure: First of all, the multilayer (including top- and bottom-electrode) is deposited without breaking the vacuum using DC magnetron sputtering. The Al_2_O_3_ tunnel barrier was fabricated by depositing Al which was afterwards partially oxidized *in-situ*, the Nb_x_O_y_ layer was deposited by reactive sputtering in an O_2_/Ar-atmosphere. Following the subsequent lift-off, the junction area was defined by etching the Au top electrode using wet etching (potassium iodide). The etched parts were then covered with thermally evaporated SiO to insulate the bottom electrode from the subsequently deposited Ti/Au-wiring to contact the top electrode.

### Electrical characterization

To measure the resistance of every single memristive device, voltage pulses were applied with the measurement setup of Fig. 4. The voltage sweeps were applied to memristive cells within the crossbar array while the current was measured simultaneously using an HP4156A source meter.

### Technical implementation

The control unit is a microcontroller (Arduino Mega 2560) managing the hardware-software interface. The microcontroller receives commands from the neurons and addresses the desired memristive devices out of the 16 × 16 crossbar array accordingly. For each row and column, the specific electrode is electrically connected using low-resistance analogue switches (DG212BDJ). By addressing the desired memristive cell, an analogue switch connects the top electrode of the device to an on-board pulse generator and the bottom electrode to a current measurement unit (see Fig. 4). Depending on the demand of the learning scheme, a read pulse or a pulse to change the conductance is applied. For this purpose, the pulse generator applies time-variable pulses of variable amplitude. The operational amplifier (LF356N) is used in transimpedance mode to measure the current with an Agilent 34411 A digital multimeter.

### Circuit layout

The electronic circuit was fabricated on a printed circuit board (PCB). The software EAGLE developed by CadSoft was employed for the circuit design. To electrically connect the memristive crossbar array to the electrical circuit, the crossbar arrays were wire bonded to custom sample holders.

## Electronic supplementary material

Supplementary Video
