# Supplementary figures and images for: Unsupervised Hebbian learning experimentally realized with analogue memristive crossbar arrays
Source: Sci Rep. 2018 Jun 11;8:8914. doi: 10.1038/s41598-018-27033-9 (PMC5995917; doi:10.1038/s41598-018-27033-9)

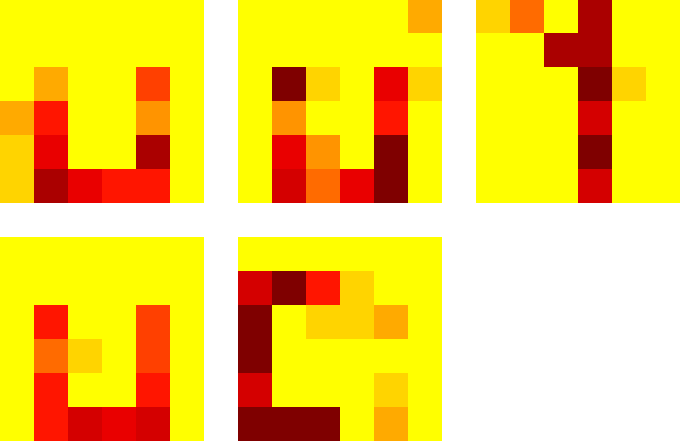

Supplement: Supplementary file 1 — Supplementary Video [file 41598_2018_27033_MOESM1_ESM.gif]
